# The net effects of medical malpractice tort reform on health insurance losses: the Texas experience

**DOI:** 10.1186/s13561-017-0174-2

**Published:** 2017-11-24

**Authors:** Patricia H. Born, J. Bradley Karl, W. Kip Viscusi

**Affiliations:** 1 Vanderbilt University Law School, 131 21st Avenue South, Nashville, TN 37203 USA; 2 Florida State University, College of Business, Tallahassee, FL USA; 3 East Carolina University, College of Business, Greenville, NC USA

**Keywords:** Medical malpractice, Health insurance, Tort reform, Liability, K13, I10, G22

## Abstract

In this paper, we examine the influence of medical malpractice tort reform on the level of private health insurance company losses incurred. We employ a natural experiment framework centered on a series of tort reform measures enacted in Texas in 2003 that drastically altered the medical malpractice environment in the state. The results of a difference-in-differences analysis using a variety of comparison states, as well as a difference-in-difference-in-differences analysis, indicate that ameliorating medical malpractice risk has little effect on health insurance losses incurred by private health insurers.

## Introduction

The motivations for reforming the medical malpractice tort environment, beginning in some states several decades ago, include assertions that limitations on liability would reduce expenditures on unnecessary health care services, specifically those services provided solely in defense of potential liability claims. In reducing defensive medicine practices, these reforms would thereby reduce overall health care costs. This assertion relies on health care providers’ responses to a reduction in perceived malpractice risk. Physicians may reduce services provided, but unless they are otherwise penalized for providing unnecessary services (e.g., through managed care plans’ profiling activities), they may be reluctant to reduce the income associated with these services. In fact, reducing providers’ expected liability could also lead to potentially *more* health care services provided, that is, a wider range of procedures supplied or more intensive treatments. If more health care services are provided, insurers will experience an increase in claims, rather than a reduction.

The markets for medical malpractice insurance and health insurance are linked via the provision of health care services. A comprehensive evaluation of reform activity in either market consequently should recognize the potential spillover effects of the reform in one market to the other. Several prior studies have addressed the relationship between a change in medical malpractice liability exposure and healthcare costs (for example, [1, 2]). A subset of these studies find evidence of provider responses to a reduction in liability within samples of patients with certain diagnoses, or among a sample of the population (for example, Medicare patients). There is little evidence of how private health insurers generally fare following reform activity. Studies that specifically consider health insurance markets examine the extent to which changes in the medical malpractice environment affect insurance premiums and coverage rates (for example, [3, 4]). However, little evidence exists regarding the extent to which a change in the medical malpractice environment, such as the implementation of a cap on noneconomic damage levels, might influence losses in the private health insurance market.

In this paper, we use the experience in Texas to evaluate the effect of medical malpractice tort reform on losses in the private health insurance market. In 2003, Texas passed a series of sweeping medical malpractice reform measures aimed at reducing the medical professional liability exposure of health care providers in the state. The enactment of these reforms provides an opportunity to examine the influence of the malpractice environment on insured health losses using a natural experiment design. Using insurance company financial data from the National Association of Insurance Commissioners (NAIC), we perform a series of Difference-in-Differences (DD) and Difference-in-Difference-in-Differences (DDD) analyses to provide evidence that tort reform in Texas had little effect on the levels of losses incurred by private health insurers on behalf of insured patients (in other words, claims for healthcare services).

More specifically, our analysis yields no support for the hypothesis that the Texas medical malpractice reforms had and substantial, persistent influence on levels of health insurance losses. We do find some evidence that suggests health insurance losses incurred by Texas insurers *increased* in the initial two years following the reform and our estimates indicate that this increase was between $400 and $500 per enrollee. However, we find no other evidence that Texas health insurer losses were affected by the reform during any other post-reform year, suggesting that the spillover effects of the reforms were, at best, short-lived. Taken in its entirety, our analysis leads us to conclude that medical malpractice reforms had little influence on reducing the cost of medical care paid for by private health insurers.

The paper proceeds as follows. In Section II we discuss the existing literature that addresses the effects of medical malpractice tort reform. We note that there are numerous studies that measure the effects of reform on the target market (that is, the influence of medical malpractice legal reforms on the profitability of medical malpractice insurers), but only a few studies evaluate the spillover to the health care environment. In Section III we derive our hypothesis, and in Section IV we describe our data. Section V presents our empirical methodology, which includes several approaches to estimate the influence of the reform using DD and DDD analyses. Section V also details our results and Section VI provides a discussion of the policy implications and a conclusion.

## Background

Numerous studies have evaluated medical malpractice liability exposure from varying perspectives. Some of the earliest research examines the extent to which demographic, medical, and legal factors influence the frequency and severity of medical malpractice insurance claims (for example, [5, 6]). Subsequent studies specifically consider the influence of tort reform on medical malpractice payments and provide evidence that tort reform measures have a non-trivial influence on medical malpractice damage awards (for example, [7]). Similarly, many studies find that the tort reform is associated with lower levels of incurred losses and lower loss ratios for medical malpractice insurance companies (for example, [8–10, 11, 12, 13, 14]). The thrust of the empirical evidence is that medical malpractice reform generates a cost-restraining effect on medical malpractice costs. Of particular relevance to our analysis is that these studies suggest that caps on non-economic damages have the greatest influence on loss levels incurred by medical malpractice insurers.

There is no consensus in the empirical literature regarding how the malpractice environment influences the actions of healthcare providers and physicians.^1^ The consequences of a state reducing malpractice liability could have a number of effects, theoretically. Providers could discontinue providing services that were solely defensive in nature. Alternatively, providers could be willing bear risky exposures that they had previously avoided. This response would be evident not only in providers offering riskier procedures, but deciding to practice in riskier specialty areas (for example, obstetrics). More far-reaching consequences include attracting of physicians from other states, thereby increasing the supply of services.

Some studies find no evidence of a relation between malpractice risk and physician behavior. For example, Sloan and Shadle [2], using survey data as well as Medicare data, conclude that medical decisions are not significantly affected by tort reform measures. Other studies provide evidence that physicians respond to higher levels of malpractice risk by practicing “positive” defensive medicine and supply additional services which are of no marginal value to the patient. For example, Kessler and McClellan [1] find that liability-reducing tort reform measures reduce the rates of defensive medicine in a sample of Medicare beneficiaries and their finding of the existence of defensive medicine practices is echoed by other studies in the literature.^2^ Still other studies provide evidence that physicians react to higher levels of malpractice risk by practicing “negative” defensive medicine whereby physicians distance themselves from certain patient interactions or, in the extreme case, withdraw from a particular healthcare market.^3^ For example, Currie and MacLeod [15] find that the implementation of caps on non-economic damages increased the frequency of C-sections among a large sample of individual births from 1989 to 2001.

The literature also provides evidence that, via its effect on physician behavior, tort reform influences private health insurance market operations.^4^ An example is Avraham and Schanzenbach [3], who use individual-level survey data from 1982 through 2007 to test the hypotheses that either 1) tort reform may reduce damage awards and defensive medicine costs or 2) tort reform may increase providers’ costs by reducing physicians’ caretaking incentives. In support for their first hypothesis, Avraham and Schanzenbach [3] find that tort reform increases insurance coverage rates. In a more recent paper, Avraham and Schanzenbach [16] find that treatment intensity for heart attack victims declines following a cap on noneconomic damages. Similarly, Avraham, Dafny, and Schanzenbach [17] find that the enactments of various tort reform measures reduce group self-insured health insurance premiums by 1 to 2%. Karl, Born, and Viscusi [18] also find that the professional liability climate has a non-trivial influence on the dollar amount of state-level health insurance losses per capita, though their results suggest that lower levels of professional liability exposure are associated with higher levels of health insurance losses.

A number of studies also specifically examine the Texas market following the state’s comprehensive medical malpractice reform in 2003.^5^ While the inefficiency of the tort system was one motivating factor for reform, the effort also recognized problems in the availability and affordability of medical malpractice coverage. It was suggested by some that “crisis” was evident in the preceding years: Texas reportedly had the lowest number of physicians per capita in the nation, and one in every four physicians had a malpractice claim filed against them each year [19].^6^ The Texas reform measures, shown in Table 1, addressed several dimensions of liability and the most striking of the reform was the measure to cap noneconomic damages. The 2003 reforms drastically changed the medical malpractice environment in the state and evidence suggests that the reform resulted in a 60% reduction in medical malpractice claims rates and a 30% reduction in payouts per claim [20, 21].

**Table 1:** Texas Reform Measures, 2003

Limits noneconomic damages to $250,000
Defendants can appeal class certification directly to the Texas Supreme Court to decide up front, not after years of costly litigation, if the plaintiff has a class action.
Law ensures that lawyers are paid in coupons if clients in a class-action suit get paid in coupons.
A new standard to ensure sued parties pay only their proportionate responsibility.
Reformed product liability laws so retailers are not liable for a manufacturer’s mistake.
Enacted liability limits for good Samaritans, volunteer firefighters, charity volunteers and teachers.
Closed loopholes that allowed trial lawyers to venue shop.

Notes: This table provides summary information regarding the tort reform measures enacted in Texas in 2003

Of the studies that specifically consider the consequences of the Texas tort reform measures enacted in 2003, the most pertinent to our study is Paik et al. [22] who examine how Medicare spending changed after the enactment of the Texas reform measures.^7^ Using both a county-level and state-level analysis, they find no evidence that Medicare spending declined after the enactment of the reform and provide a degree of evidence that spending increased following the 2003 reforms. The analysis of Paik et al. [22] is insightful because it suggests that physicians in Texas did not alter defensive medicine practices in a way that led to lower health insurance cost. In fact, the reform in Texas may have altered provider behavior in ways that *increase* healthcare costs, which is the opposite effect that many proponents of the Texas tort liability reform had predicted.

In summary, there exists considerable evidence that medical malpractice reform measures reduce medical malpractice awards and also the losses incurred by medical malpractice insurance companies. There is also disagreement in the literature regarding the extent to which medical malpractice reforms have any meaningful influence on the provider-patient interaction. However, some studies provide evidence that tort reform’s influence on provider behavior ultimately leads to consequences for health insurance markets but, again, there is no general consensus in the literature as to if and how tort reforms influence insured loss levels in health insurance markets.

## Methods

### Hypothesis development

Theory and empirical evidence to date suggest that the indirect effects of tort reform on health insurance costs are ambiguous. We develop our main hypothesis under the assumption that risk of a medical malpractice lawsuit influences the nature of the medical care given by health care providers and, more broadly, the provider marketplace. Prior to reform, a state’s medical malpractice insurance regulation and unique demographic characteristics are associated with a level of medical malpractice insurance claims which reflects, among other things, the litigiousness of the population and expertise of health care providers. We hypothesize that providers perceive their risk of being sued for medical malpractice in a rational manner, guided by their prior experience, information about malpractice claims being brought against other providers, or the cost of medical malpractice insurance.^8^ Assuming that the medical malpractice environment affects the expected liability costs, there will be an incentive for medical malpractice providers to take actions to reduce exposure to risk. For example, a provider who perceives an increase in liability exposure could order more tests for insured patients, see fewer patients with specific health issues, or even exit the geographic market altogether. These behavioral changes will generate a change in levels of health insurance claims, and we might expect to find a significant relationship between changes in the legal environment for medical malpractice and losses incurred by health insurers. However, since providers may respond in ways that either increase health care costs or reduce health care costs, the direction of this relationship, when evaluated in the aggregate, is ambiguous. To the extent that changes in behavior might, in effect, all cancel each other out in the aggregate, we provide the following null hypothesis:

**H**_**o**_ **:** *Liability-reducing* r*eform in the medical malpractice market has no effect on the level of health insurance losses.*

If we are able to reject the null hypothesis, then we find in favor of an alternative hypothesis that medical malpractice reform leads to changes in provider behavior that significantly increases or decreases health insurance losses. To the extent that providers do not instantaneously comprehend the consequences of the reforms at the time of enactment, the effect on the health insurance market may be potentially delayed. However, efforts to over-treat for defensive reasons will result in an increase in health insurance losses while efforts to avoid certain patients will result in a reduction in health insurance losses. We note that rejection of the null hypotheses could also result from changes in provider behavior outside of simply interacting with the patient. Reforms could lead to an expansion in the number of physicians in the state and the supply of medical care. Medical malpractice market reforms also could influence the nature of rents demanded by physicians from health insurance companies, thereby potentially influencing health insurance losses without changing the nature of provider-patient interactions. As such, evidence on the validity of our hypothesis will not evaluate the specific nature of a medical professionals’ behavior changes surrounding medical malpractice reforms, but rather the ultimate effect of the changes on health insurance losses.

Examining the experience of the private health insurers in Texas before and after the malpractice reform effort would provide evidence on whether malpractice reforms have implications for health insurance markets as well as the direction of these effects. Specifically, if the reforms passed in Texas had no effect on provider behavior, then we would expect the levels of health insurance losses incurred by Texas health insurers to be equal before and after the reform. Such a result would provide support for our null hypothesis. Alternatively, if the Texas reforms altered physician behavior in a way that resulted in either higher or lower levels of health insurance losses, then we would expect levels of health insurance losses incurred by health insurers in Texas before the reforms to differ from the levels after the implementation of the reforms. Such a result would support our alternative hypothesis that the ramifications of medical malpractice reforms for health insurance are consequential.

### Data

We identify several sources of data to test our hypothesis. Data on state tort reform measures comes from the American Tort Reform Association (ATRA) and the Database of State Tort Law Reforms [23]. State demographic data, added to the analysis for a further robustness check, is obtained from the Centers for Disease Control (CDC) and the U.S. Census Bureau. “Health Status” is a variable provided by the CDC that indicates the overall health status of a given state in a given year and is increasing in good health. “Dependents” is the number of persons under the age of 18 per capita in a given state in a given year. “Females” is the proportion of a state’s population that is female in a particular year. “Median Income” is the median income level for residents of a given state during a given year. “Unemployment Rate” is the proportion of a particular state’s available workforce that is not employed in a given year.

Testing of our hypothesis also requires state-specific data pertaining to health insurance losses. We use insurer financial data from the state pages of the National Association of Insurance Commissioners (NAIC) statutory filings for the years 2001 through 2010.^9^ This dataset provides the most complete and comprehensive database of private health insurance losses.^10^ We then apply several filters to this raw dataset in order to screen out insurers that do not have a significant level of business in a given state.^11^ Since we are interested in examining the extent to which loss levels incurred by health insurers changed following the Texas reform, it would be inappropriate to include firms that enter a state market after the reform. As such, if insurer *i* does not operate in state *j* from 2001 to 2003, we remove that insurer-state observation for all future years.^12^

To test our hypothesis relating to the influence of tort reform on health insurance losses, we use the NAIC data to calculate health insurance losses per enrollee (LPE). This variable is defined as total health insurance losses incurred for insurer *i* in state *j* during year *t* scaled by total health enrollees for insurer *i* in state *j* during year *t* and is ideal for our analysis because it provides a standardized metric of health insurance losses which facilitates comparison across all firms.^13^ In all tables and figures presented in this analysis, LPE is always expressed as scaled by $1000 for ease of formatting.

Our analysis focuses on insurers operating in Texas, New Jersey, Colorado, and three additional subsamples of states that did not enact significant medical malpractice reforms during our sample period. Table 2 provides summary statistics of health insurance LPE, scaled by $1000, for the insurers operating in these states from 2001 to 2010 in terms of 2010 dollars.^14^ The table indicates that LPE generally increased over our sample period in all state samples and suggests that healthcare costs are rising, in general. Summary inspection of the Texas data, in particular, indicates that insurers’ mean LPE increased by roughly about $1000 from the beginning to the end of our sample period. However, there is no obvious break in this trend surrounding the enactment of the Texas reforms, which is consistent with our null hypothesis.

**Table 2:** Health Insurance Losses per Enrollee for Different Samples

Health Insurance Losses per Enrollee									
Panel A									
	Texas			New Jersey			Colorado		
Year	Mean	St. Dev.	Insurers	Mean	St. Dev.	Insurers	Mean	St. Dev.	Insurers
2001	1.56	1.85	45	1.78	1.08	18	1.78	1.21	18
2002	1.53	1.49	41	1.45	1.48	34	1.99	1.36	20
2003	1.88	1.79	43	1.41	1.53	34	1.96	1.40	18
2004	1.99	2.09	39	1.51	1.75	34	2.02	1.47	18
2005	2.04	2.22	38	1.71	2.38	34	2.22	1.60	18
2006	1.99	2.23	38	1.39	1.56	31	2.33	1.71	18
2007	2.25	2.52	38	1.47	1.74	30	2.45	1.85	18
2008	2.22	2.62	37	1.50	1.82	30	2.30	1.97	16
2009	2.52	3.00	34	1.71	2.07	29	2.57	2.30	16
2010	2.10	2.67	32	1.72	2.29	28	2.53	2.38	15
Panel B									
	41 State Subsample			18 State Subsample			9 State Subsample		
Year	Mean	St. Dev.	Insurers	Mean	St. Dev.	Insurers	Mean	St. Dev.	Insurers
2001	1.95	3.99	545	1.90	1.27	223	1.94	1.26	96
2002	1.87	1.70	588	2.02	1.72	241	2.07	1.70	108
2003	1.96	1.72	587	2.16	2.01	248	1.98	1.69	110
2004	2.14	1.89	554	2.38	2.17	234	2.14	1.79	103
2005	2.13	1.79	533	2.31	1.89	222	2.21	1.93	96
2006	2.30	1.99	520	2.57	2.26	218	2.53	2.34	95
2007	2.46	2.09	491	2.71	2.30	207	2.64	2.40	92
2008	2.56	2.27	483	2.92	2.57	205	2.67	2.45	89
2009	2.73	2.46	463	3.15	2.83	196	2.90	2.80	88
2010	2.76	2.55	440	3.19	2.88	191	2.97	2.88	86

Notes: This table provides summary information regarding health insurance firms’ Losses per Enrollee (LPE) for each of the subsample of firms used in our analysis, during each year of our sample period. LPE is defined as the dollar amount of health insurance losses incurred by a given insurer, in a given state, during a given year, scaled by the number of plan enrollees for a given insurer, in a given state, during a given year. LPE is also scaled by 1000. Panel A provides information pertaining to LPE for a subsample of insurers operating in Texas, New Jersey, or Colorado. Panel B provides information pertaining to LPE for three subsamples of insures operating in states identified by Paik et al. [24]. “Mean” refers to the mean value of LPE, “St. Dev.” refers to the standard deviation of LPE, and “Insurers” refers to the number of insurers (observations)

**Fig. 1:**
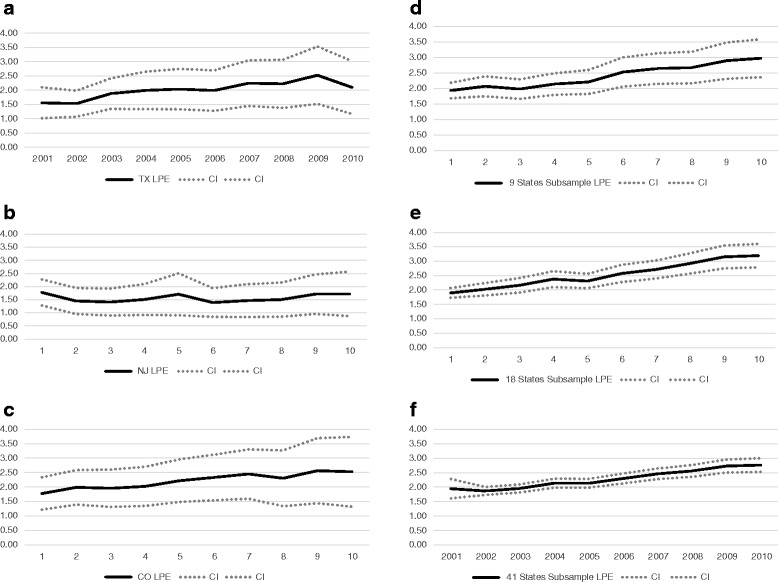
**a** Trends in health insurance losses per enrollee (LPE) – Texas. **b** Trends in health insurance losses per enrollee (LPE) – New Jersey.**c** Trends in health insurance losses per enrollee (LPE) – Colorado. **d** Trends in health insurance losses per enrollee (LPE) – 9 State Subsample. **e** Trends in health insurance losses per enrollee (LPE) – 18 State Subsample. **f** Trends in health insurance losses per enrollee (LPE) - – 41 State Subsample.Notes: These figures display trends in health insurance firms’ Losses per Enrollee (LPE), for each of the subsample of firms used in our analysis during our sample period. LPE is defined as the dollar amount of health insurance losses incurred by a given insurer, in a given state, during a given year, scaled by the number of plan enrollees for a given insurer, in a given state, during a given year. LPE is also scaled by 1000

Figure 1a – 1f show the mean LPE, and the 95% confidence interval around the mean for the different samples of insurers in our analysis across our sample period. The figures reinforce our observations in the summary data. The gradual upward trend in Texas LPE is easily observable and, with the exception of New Jersey, largely mirrors the trends observed in the other non-reforming states. However, the figure does highlight a relatively sudden increase in LPE in Texas in 2003 – the year the reforms were enacted – relative to 2002. The magnitude of this increase in mean LPE is approximately $300 and may suggest that reforms had the initial effect of increasing health insurance losses incurred by Texas insurers. We investigate this possibility in more detail in the ensuing sections.

### Difference-in-differences analysis

The dramatic overhaul of Texas’ medical professional liability climate in 2003 resulting from the enactment of medical malpractice reforms presents an ideal setting for testing our hypothesis using a natural experiment design.^15^ If, as our alternative hypothesis predicts, the change in the medical malpractice environment led to changes in the way medical providers behave in the healthcare market, which ultimately led to changes in health insurance losses, then we would not expect health insurance loss levels before the reform to equal loss levels after the reform. Further, since the reform measures only apply to the legal environment in Texas after the implementation of the new law, we would not expect the law passed in Texas to have an influence on the insurance markets of other states pre- or post-Texas reform. Therefore, comparing the difference in Texas health insurance losses levels pre- and post-Texas reform to the difference in the health insurance losses levels pre- and post- the Texas reform of a state unaffected by the losses allows us to isolate the direct influence of the tort reform measures on the health insurance market in Texas.

For robustness in the DD, we first identify insurers operating in two different non-treated states – New Jersey and Colorado, and perform two separate DD analyses. Neither state had major upheaval in the health insurance marketplace (such as health insurance reforms) in the time closely preceding and following the implementation of the Texas tort reforms. Further, neither state enacted any major medical malpractice insurance reforms during the time of the Texas tort reforms. Of note is that Colorado had several tort reform measures in place prior to 2003, including caps on non-economic damages (enacted in 1987), while New Jersey had relatively few tort reform measures in place and no caps on non-economic damages.

Following Paik et al. [22], we also identify three additional non-treated subsamples, comprised of insurers operating in states unaffected by tort reforms during our sample period. The first subsample consists of insurers operating in the 41 states that did not enact a major tort reform from 2001 to 2010.^16^ The second subsample consists of insurers operating in the 18 states that never enacted a cap on non-economic damages or total damages during the sample period.^17^ The third subsample consists of insurers operating in nine states that did not enact a cap on damages and, as suggested by Paik et al. [22], are similar to Texas both geographically and culturally.^18^ Using the same non-treated states as Paik et al. [22] adds another element of robustness to our individual state comparisons and allows us to consider their conclusions in the context of private health insurance markets.^19^

In theory, implementation of the DD analysis involves comparing the difference in mean health insurance LPE between insurers operating in Texas and insurers in the non-treated samples before the enactment of the Texas reform. This difference is then compared to the difference in mean health insurance LPE between insurers operating in Texas and insurers in the non-treated samples after the Texas reform. While the Texas reforms went into effect in the latter part of 2003, their first full year of implementation was 2004. As a result, our DD analysis considers how losses changed in 2004 and onward relative to 2003 and before.

In practice, the DD analysis is implemented using a regression framework.^20^ We estimate several unique model specification that take the general form of the following OLS model:

1 $$ {LPE}_{it}=a+{\beta}_1{Treat}_{it}+{\beta}_2\mathit{\operatorname{Re}}{form}_t+{\beta}_3{Treat}_{it}\ast \mathit{\operatorname{Re}}{form}_t+{\varepsilon}_{it} $$

where.

*Treat* = a dummy variable indicating insurer *i* is a member of the treatment group in year *t* and captures differences between the treatment and control group. In our analysis, *Treat* is equal to one for insurers operating in Texas and zero for insurers operating in the other non-treated states described previously;

*Reform* = a dummy variable equal to one if the year is greater than or equal to 2004 and 0 if the year is less than 2004; and.

Treat*Reform = a dummy variable equal to one for insurers that are members of the treatment group in the years after the enactment of the tort reforms.

The coefficient on Treat*Reform, *β*_3_, is the DD estimator. Formally,

$$ {\beta}_3=\left({\overline{LPE}}_{Treat=1,\mathit{\operatorname{Re}} form=1}-{\overline{LPE}}_{Treat=1,\mathit{\operatorname{Re}} form=0}\right)-\left({\overline{LPE}}_{Treat=0,\mathit{\operatorname{Re}} form=1}-{\overline{LPE}}_{Treat=0,\mathit{\operatorname{Re}} form=0}\right). $$

The numerical value of this coefficient is the difference in the differences of mean health insurance LPE in Texas and the control state before and after the implementation of the reforms. The t-test of the coefficient indicates if the difference-in-difference estimate is statistically significant. A statistically insignificant *β*_3_ would prevent us from rejecting the null hypothesis that the Texas reforms influenced physician behavior in a way that spilled over into the health insurance marketplace. A statistically significant and positive (negative) *β*_3_ would provide support for our alternative hypothesis that the enactment of the Texas tort reforms influenced physician behavior in a way that, in the aggregate, increased (decreased) health insurance losses.

### Difference-in-difference-in-differences analysis

In an effort to provide further evidence on the validity of our hypothesis, we employ a difference-in-difference-in-differences (DDD) analysis where we include, as an additional control group, a sub-sample insurers operating in lines of business not related to health insurance or medical malpractice markets. The identification assumptions of the DDD are more robust than that of a DD analysis and helps to confirm the findings of the previous section. In particular, a DDD strategy controls for the potentially confounding trend of changes in health insurance losses over time that are not related to medical malpractice reform^21^ and also controls for the confounding effects of state-specific factors that affect insurance losses, generally. As such, the DDD framework improves on the shortcomings of the DD analysis by controlling for a broad set of other influences. If our results are robust to a DDD analysis, this would suggest that our results are not due to spurious developments in the state’s health insurance environment.

To implement the DDD, we select as the additional control group a subsample of insurers operating in private passenger automobile physical damage insurance in Texas, New Jersey, Colorado, and the three multi-state subsamples identified by Paik et al. [22].^22^ We quantify the losses incurred by these insurers in the given states as losses per automobile (LPA), calculated as the amount of private passenger automobile physical damage losses incurred by insurer *i* in state *j* during year *t* scaled by a weighted measure of the number of automobiles insured by insurer *i* in state *j* during year *t*.^23^ We then compare the difference-in-differences between LPE and LPA in Texas pre and post Texas tort reform with the difference-in-differences between LPE and LPA in the control state(s) pre and post Texas tort reform.

In practice, the DDD analysis is implemented using a regression framework. We estimate several unique model specifications that take the general form of the following OLS model:

2 $$ {Losses}_{it}={a}_i+{\beta}_1 Treat+{\beta}_2 Control+{\beta}_3 Treat\ast Control+{\beta}_4\mathit{\operatorname{Re}} form+{\beta}_5 Treat\ast \mathit{\operatorname{Re}} form+{\beta}_6 Control\ast \mathit{\operatorname{Re}} form+{\beta}_7 Treat+ Control\ast \mathit{\operatorname{Re}} form+{\varepsilon}_{it} $$

where.

*Losses* = insurer *i*’s LPE if the insurer is a health insurer or insurer *i*’s LPA if the insurer is an auto insurer in a given state in a given year;

*Treat* = a dummy variable indicating insurer *i* is a member of the treatment group in year *t* and captures differences between the treatment and control group. In our analysis, *Treat* is equal to one for insurers operating in Texas and zero for insurers operating in the other states described previously;

*Control* = a dummy variable indicating insurer *i* is a health insurer in year *t* and captures the effects that the insurance market, in general, may have on health insurance losses levels. In our analysis, *Control* is equal to one if the insurer is operating in health insurance lines and equal to zero if the insurer is operating in automobile insurance lines in a given state in a given year;

*Reform* = a dummy variable equal to one if the year is greater than or equal to 2004 and 0 if the year is less than 2004; and.

Treat*Control*Reform = a dummy variable equal to one if insurer *i* is a health insurer operating in a non-treated state in year 2004 or later.

The coefficient on Treat*Control*Reform, *β*_7_, is the difference-in-differences-in-differences estimator. The numerical value of this coefficient is the difference-in-differences-in-differences of mean LPE and LPA in Texas and the control state before and after the implementation of the reforms. The t-test of the coefficient indicates if the DDD is statistically significant. A statistically insignificant *β*_7_ would prevent us from rejecting the null hypothesis that the Texas reforms influenced physician behavior in a way that spilled over into the health insurance marketplace. A statistically significant and positive (negative) *β*_7_ would provide support for our alternative hypothesis that the enactment of the Texas tort reforms influenced physician behavior in a way that, in the aggregate, increased (decreased) health insurance losses.

## Results and discussion

Table 3 displays the results of estimating eq. 1 for five distinct model specifications, where each specification differs only by the sample of insurers designated as non-treated. For all specifications, we cluster standard errors at the firm level. As shown in the table, none of the coefficients on the DD estimator are statistically significant at conventional levels. This result suggests that the mean change in Texas health insurers’ LPE was not statistically different from the mean change in non-reform state health insurers’ LPE from in the years 2004–2010 relative to 2001–2003. This evidence is consistent with our null hypothesis that the Texas tort reform efforts had no spillover effects that substantially influenced the losses incurred by health insurers.

**Table 3:** Basic Difference-in-Differences Regression Analyses of Texas Reforms

	New Jersey	Colorado	41 State Subsample	18 State Subsample	9 State Subsample
DD Estimator	0.4276	0.0687	−0.0039	−0.1983	−0.0706
	[0.268]	[0.304]	[0.243]	[0.251]	[0.269]
Treatment Dummy	0.1557	−0.2526	−0.2653	−0.3743	−0.3398
	[0.319]	[0.342]	[0.233]	[0.243]	[0.262]
Reform Dummy	0.0689	0.4278**	0.5004***	0.6948***	0.5671***
	[0.138]	[0.209]	[0.091]	[0.114]	[0.150]
Constant	1.5024***	1.9107***	1.9234***	2.0323***	1.9979***
	[0.232]	[0.282]	[0.113]	[0.144]	[0.175]
Observations	687	560	5589	2570	1348
R-squared	0.0182	0.0137	0.0110	0.0260	0.0202

Notes: This table presents the results of several difference-in-differences analyses obtained using the regressions described generally in eq. 1. The dependent variable, Losses per Enrollee (LPE), is defined as the dollar amount of health insurance losses incurred by a given insurer, in a given state, during a given year, scaled by the number of plan enrollees for a given insurer, in a given state, during a given year. LPE is also scaled by 1000. In the table, “DD estimator” is the difference-in-differences estimator, “Treatment dummy” indicates firms operating in Texas, and “Reform Dummy” indicates years following the enactment of the Texas reform measures. Each column of output represents a separate analysis that differs only by the subsample of firms used as non-treated groups. Clustered standard errors are presented in parentheses and ***indicates *p* < 0.01, and **indicates *p* < 0.05

The results presented in Table 4 confirm that, with one exception, the results displayed in Table 3 are not sensitive to the inclusion of state-level demographic control variables. The exception is that we find that mean changes in LPE were *larger* in for Texas insurers in the years following the reform relative to their New Jersey counterparts. The magnitude of this coefficient, which is significant at the 10% level, indicates that, relative to New Jersey, insurers operating in Texas experience an increase in LPE of approximately $519, on average, in the years following the reforms. This result is consistent with our alternative hypothesis and other findings in the literature (e.g., [22]) that the reforms in Texas actually bent the healthcare cost curve upward. However, in light of the fact that the DD estimator is not statistically significant in the other model specifications, the results of the New Jersey specification do not provide compelling support for the rejection of our null hypothesis.

**Table 4:** Difference-in-Differences Regression Analyses of Texas Reforms Including Full Variable Set

	New Jersey	Colorado	41 State Subsample	18 State Subsample	9 State Subsample
DD Estimator	0.5193*	0.1734	0.0566	−0.1421	−0.1018
	[0.292]	[0.291]	[0.249]	[0.262]	[0.276]
Treatment Dummy	−0.5373	−0.1091	0.2461	0.1101	−0.3308
	[0.748]	[0.578]	[0.331]	[0.545]	[0.755]
Reform Dummy	0.1043	0.2086	0.3786***	0.5063**	0.6322**
	[0.163]	[0.139]	[0.130]	[0.199]	[0.247]
Health Status	−0.0056	0.0253	−0.0285	−0.0319	−0.0535
	[0.037]	[0.043]	[0.027]	[0.042]	[0.038]
Dependents	0.2297	−0.0202	−0.1281***	−0.0814	0.0458
	[0.215]	[0.200]	[0.048]	[0.121]	[0.198]
Females	−0.0646	−0.0173	−0.0125	0.0355	0.0006
	[0.063]	[0.118]	[0.073]	[0.085]	[0.109]
Median Income	0.0000	0.0000	−0.0000	0.0000	−0.0000
	[0.000]	[0.000]	[0.000]	[0.000]	[0.000]
Unemployment Rate	0.0774***	0.0347	0.0607***	0.0714**	0.0600*
	[0.023]	[0.031]	[0.021]	[0.029]	[0.033]
Constant	−1.9597	1.2360	6.0315	1.6430	1.6183
	[5.237]	[7.573]	[4.276]	[5.848]	[9.012]
Observations	687	560	5589	2570	1348
R-squared	0.0202	0.0161	0.0197	0.0370	0.0276

Notes: This table presents the results of several difference-in-differences analyses obtained using the regressions described generally in eq. 1. The dependent variable, Losses per Enrollee (LPE), is defined as the dollar amount of health insurance losses incurred by a given insurer, in a given state, during a given year, scaled by the number of plan enrollees for a given insurer, in a given state, during a given year. LPE is also scaled by 1000. “DD estimator” is the difference-in-differences estimator, “Treatment dummy” indicates firms operating in Texas, and “Reform Dummy” indicates years following the enactment of the Texas reform measures. “Health status”, “Dependents”, “Females”, “Median income” and “Unemployment rate” are all state-level demographic control variables previously described. Each column of output represents a separate analysis that differs only by the subsample of firms used as non-treated groups. Clustered standard errors are presented in parentheses and ***indicates *p* < 0.01, **indicates *p* < 0.05, and *indicates *p* < 0.1

As a robustness exercise, we compare differences in losses levels in 2002 to differences in losses levels in 2004, 2005, 2006, 2007, 2008, 2009, and 2010. These estimates help to illustrate the significance and magnitude of the difference in LPE in the year immediately preceding the enactment of the reforms, to each individual year following the reform. The results of this year-by-year DD analysis for the insurers operating in the subsample of 9 states as the control group are given in Table 5.^24^ For the sake of brevity, we discuss the results of the other subsamples, where relevant, instead of reporting the full year-by-year analysis for all subsamples.^25^ As shown in the table, the DD estimator is statistically significant at the 10% level only in the 2002–2004 period. This result indicates that, relative to the year 2002, mean LPE increased at a greater rate for Texas insurers than the non-Texas insurers in the 9 state subsample for the year 2004 through 2010. The magnitude of the coefficient suggest the change in LPE experienced by Texas health insurers was approximately $390 greater than their counterparts in the 9 state subsample.

**Table 5:** Difference-in-Differences Regression Analyses of Texas Reforms for Nine State Sample and Multiple Time Periods

	2002–2004	2002–2005	2002–2006	2002–2007	2002–2008	2002–2009	2002–2010
DD Estimator	0.3914*	0.3710	0.0004	0.1473	0.0909	0.1681	−0.3316
	[0.235]	[0.255]	[0.260]	[0.323]	[0.342]	[0.426]	[0.381]
Treatment Dummy	−0.5408*	−0.5408*	−0.5408*	−0.5408*	−0.5408*	−0.5408*	−0.5408*
	[0.293]	[0.293]	[0.293]	[0.293]	[0.293]	[0.293]	[0.293]
Reform Dummy	0.0739	0.1404	0.4590***	0.5727***	0.6043***	0.8266***	0.9023***
	[0.117]	[0.131]	[0.169]	[0.190]	[0.189]	[0.233]	[0.255]
Constant	2.0696***	2.0696***	2.0696***	2.0696***	2.0696***	2.0696***	2.0696***
	[0.201]	[0.201]	[0.201]	[0.201]	[0.201]	[0.201]	[0.201]
Observations	291	283	282	279	275	271	267
R-squared	0.0130	0.0143	0.0276	0.0321	0.0333	0.0438	0.0502

Notes: This table presents the results of several difference-in-differences analyses obtained using the regressions described generally in eq. 1 and only using the subsample of firms operating in 9 states as the non-treated group. The dependent variable, Losses per Enrollee (LPE), is defined as the dollar amount of health insurance losses incurred by a given insurer, in a given state, during a given year, scaled by the number of plan enrollees for a given insurer, in a given state, during a given year. LPE is also scaled by 1000. In the table, “DD estimator” is the difference-in-differences estimator, “Treatment dummy” indicates firms operating in Texas, and “Reform Dummy” indicates years following the enactment of the Texas reform measures. Each column of output represents a separate analysis that compares LPEs in the year 2002 to a given, single year in the future. Clustered standard errors are presented in parentheses and ***indicates *p* < 0.01, and *indicates *p* < 0.1

In unreported analysis, we also find changes in Texas LPE in 2004, relative to 2002, were greater than the changes experienced by health insurers operating in New Jersey and Colorado over the same period. However, regardless of the sample examined, we find no other evidence that changes in Texas LPE in any ensuing year (i.e. 2005 to 2010) were significantly greater than those experienced by insurers operating in other states. Taken together, this year-by-year DD analysis suggests that the spillover effects of the Texas reforms into the health insurance market were, at best, short-lived and influenced Texas health insurers only during 2004. While this result does favor our alternative hypothesis, the evidence is weak and does not provide compelling evidence that the Texas reforms had a long-lasting and substantial effect on the health insurance market.

Considered in their entirety, the results presented in Tables 3 through 5 provide little support for the hypothesis that the Texas reforms had a significant influence on the health insurance market – the vast majority of our model specifications fail to find a significant change in Texas LPE after the enactment of tort liability reforms. In the few instances we do find a statistically significant spillover effect, our estimates suggest the reforms had the effect of *increasing* LPE. However, in these cases, the results are not robust across all subsample analyses and/or the effect is short-lived and we therefore are unable to reject null hypotheses based on the evidence in the DD analysis. In the ensuing subsection, we explore the robustness of our findings by extending our DD analysis to control for other potential confounding factors.

Table 6 displays the results of estimating eq. 2, our DDD model, for five distinct model specifications, where each specification differs only by the sample of insurers designated as non-treated.^26^ The DDD estimator is statistically insignificant in all but one of the five model specifications which provides little support for the hypothesis that the Texas liability reforms had any meaningful impact on losses incurred by health insurers operating in Texas. However, the DDD estimator is statistically significant at the 5 % level when the New Jersey insurer subsample is used and the magnitude of the coefficient indicates the net increase in LPE incurred by Texas insurers in the post-reform time period was approximately $620. The results in Table 7 further suggest that our DDD analysis is robust to the addition of several state-level demographic control variables.^27^ The DDD analysis, therefore, provides little evidence in favor of our alternative hypothesis.

**Table 6:** Basic Differences-in-Differences-in-Differences Regression Analyses of Texas Reforms

	New Jersey	Colorado	41 State Subsample	18 State Subsample	9 State Subsample
DDD Estimator	0.6191**	0.2000	0.1505	−0.0424	0.0821
	[0.270]	[0.305]	[0.245]	[0.253]	[0.271]
Treatment Dummy	0.3992***	0.2950***	0.3064***	0.3221***	0.2984***
	[0.038]	[0.039]	[0.037]	[0.037]	[0.038]
Control Dummy	1.2416***	1.5457***	1.5698***	1.6944***	1.6363***
	[0.231]	[0.280]	[0.113]	[0.143]	[0.174]
Control*Treatment	−0.2435	−0.5476	−0.5717**	−0.6964***	−0.6382**
	[0.320]	[0.342]	[0.236]	[0.246]	[0.264]
Reform Dummy	−0.0138	−0.0741***	−0.0509***	−0.0494***	−0.0527***
	[0.016]	[0.012]	[0.005]	[0.006]	[0.008]
Treatment*Reform	−0.1916***	−0.1312***	−0.1544***	−0.1560***	−0.1527***
	[0.041]	[0.039]	[0.037]	[0.037]	[0.037]
Control*Reform	0.0827	0.5019**	0.5513***	0.7442***	0.6198***
	[0.138]	[0.208]	[0.091]	[0.114]	[0.150]
Constant	0.2608***	0.3650***	0.3536***	0.3379***	0.3616***
	[0.011]	[0.011]	[0.005]	[0.006]	[0.008]
Observations	2447	2873	42,436	21,281	11,603
R-squared	0.2338	0.3113	0.3469	0.4252	0.3794

Notes: This table presents the results of several difference-in-differences-in-differences analyses obtained using the regressions described generally in eq. 2. The dependent variable, Losses per Enrollee (LPE), is defined as the dollar amount of health insurance losses incurred by a given insurer, in a given state, during a given year, scaled by the number of plan enrollees for a given insurer, in a given state, during a given year. LPE is also scaled by 1000. “DDD estimator” is the difference-in-differences-in-differences estimator, “Treatment dummy” indicates firms operating in Texas, “Reform Dummy” indicates years following the enactment of the Texas reform measures, “Control dummy” indicates health insurers, “Control*Treatment” is the interaction of Control dummy and Treatment dummy, “Treatment*Reform” is the interaction of Treatment dummy and Reform dummy, and “Control*Reform” is the interaction of Control dummy and Reform dummy. Each column of output represents a separate analysis that differs only by the subsample of firms used as non-treated groups. Clustered standard errors are presented in parentheses and ***indicates p < 0.01, **indicates p < 0.05, and *indicates *p* < 0.1

**Table 7:** Difference-in-Differences-in-Differences Regression Analyses of Texas Reforms Including Full Variable Set

	New Jersey	Colorado	41 State Subsample	18 State Subsample	9 State Subsample
DDD Estimator	0.6221**	0.2013	0.1516	−0.0432	0.0817
	[0.269]	[0.305]	[0.245]	[0.253]	[0.271]
Treatment Dummy	0.1862	0.0750	0.3041***	0.2471***	0.0820
	[0.215]	[0.118]	[0.044]	[0.061]	[0.077]
Control Dummy	1.2415***	1.5460***	1.5711***	1.6950***	1.6355***
	[0.232]	[0.280]	[0.114]	[0.143]	[0.174]
Control*Treatment	−0.2435	−0.5480	−0.5730**	−0.6969***	−0.6375**
	[0.321]	[0.342]	[0.236]	[0.246]	[0.263]
Reform Dummy	−0.0218	−0.0665***	−0.0412***	−0.0263	0.0145
	[0.052]	[0.023]	[0.013]	[0.017]	[0.023]
Treatment*Reform	−0.1455***	−0.1113***	−0.1523***	−0.1608***	−0.1607***
	[0.052]	[0.040]	[0.037]	[0.038]	[0.038]
Control*Reform	0.0805	0.5010**	0.5507***	0.7452***	0.6208***
	[0.136]	[0.208]	[0.091]	[0.114]	[0.150]
Health Status	0.0124	0.0149	−0.0033	−0.0060	−0.0051
	[0.012]	[0.010]	[0.003]	[0.005]	[0.004]
Dependents	0.0529	0.0553	0.0003	0.0226*	0.0641***
	[0.071]	[0.040]	[0.005]	[0.013]	[0.021]
Females	−0.0160	0.0069	0.0009	−0.0017	−0.0223*
	[0.025]	[0.024]	[0.010]	[0.011]	[0.012]
Median Income	0.0000	0.0000	−0.0000	−0.0000	−0.0000
	[0.000]	[0.000]	[0.000]	[0.000]	[0.000]
Unemployment Rate	0.0215***	0.0178***	0.0101***	0.0095**	0.0077*
	[0.008]	[0.007]	[0.003]	[0.004]	[0.004]
Constant	−0.7595	−1.9630	0.4092	−0.0350	0.0713
	[1.976]	[1.368]	[0.571]	[0.674]	[0.954]
Observations	2447	2873	42,436	21,281	11,603
R-squared	0.2342	0.3118	0.3472	0.4260	0.3831

Notes: This table presents the results of several difference-in-differences-in-differences analyses obtained using the regressions described generally in eq. 2. The dependent variable, Losses per Enrollee (LPE), is defined as the dollar amount of health insurance losses incurred by a given insurer, in a given state, during a given year, scaled by the number of plan enrollees for a given insurer, in a given state, during a given year. LPE is also scaled by 1000. In the table, “DDD estimator” is the difference-in-differences-in-differences estimator, “Treatment dummy” indicates firms operating in Texas, “Reform Dummy” indicates years following the enactment of the Texas reform measures, “Control dummy” indicates firms operating as health insurers, “Control*Treatment” is the interaction of Control dummy and Treatment dummy, “Treatment*Reform” is the interaction of Treatment dummy and Reform dummy, and “Control*Reform” is the interaction of Control dummy and Reform dummy. “Health status”, “Dependents”, “Females”, “Median income” and “Unemployment rate” are all state-level demographic control variables previously described. Each column of output represents a separate analysis that differs only by the subsample of firms used as non-treated groups. Clustered standard errors are presented in parentheses and ***indicates *p* < 0.01, **indicates *p* < 0.05, and *indicates *p* < 0.1

In Table 8, we provide the results of a DDD analysis where LPE in 2002 is compared to the years 2004 through 2010 in an effort to illustrate the significance and magnitude of difference in LPE in the year immediately preceding the enactment of the reforms, to each individual year following the reform.^28^ The table indicates that, relative to the non-treated group of insurers operating in the nine-state subsample, mean LPE in Texas were higher and statistically different from zero in the first two years following the reform. In particular, the magnitude of the DDD estimator coefficient suggests that the net increase in mean LPE for Texas insurers in 2004, relative to 2002, was approximately $490 and this same increase was approximately $435 in 2005 relative to 2002. Again, this evidence suggests that, at best, the spillover effect of the Texas reforms on the health insurance market was short-lived. In unreported analysis using the New Jersey insurers, Colorado insurers, and the 41 state subsample of insurers, we find further evidence of statistically significant increases in mean LPE in the initial year or two following the reform that do not persist to future years.

**Table 8:** Differences-in-Differences-in-Differences Regression Analyses of Texas Reforms for Nine State Sample and Multiple Time Periods

	2002–2004	2002–2005	2002–2006	2002–2007	2002–2008	2002–2009	2002–2010
DDD Estimator	0.4919**	0.4347*	0.0733	0.2393	0.0666	0.2097	−0.1943
	[0.235]	[0.255]	[0.260]	[0.322]	[0.343]	[0.424]	[0.379]
Treatment Dummy	0.2154***	0.2154***	0.2154***	0.2154***	0.2154***	0.2154***	0.2154***
	[0.023]	[0.023]	[0.023]	[0.023]	[0.023]	[0.023]	[0.023]
Control Dummy	1.7160***	1.7160***	1.7160***	1.7160***	1.7160***	1.7160***	1.7160***
	[0.199]	[0.199]	[0.199]	[0.199]	[0.199]	[0.200]	[0.200]
Control*Treatment	−0.7563***	−0.7563***	−0.7563***	−0.7563***	−0.7563***	−0.7563***	−0.7563***
	[0.292]	[0.292]	[0.292]	[0.292]	[0.292]	[0.292]	[0.292]
Reform Dummy	−0.0404***	−0.0584***	−0.0429***	−0.0415***	−0.0299**	−0.0555***	−0.0442***
	[0.008]	[0.007]	[0.007]	[0.007]	[0.014]	[0.008]	[0.007]
Treatment*Reform	−0.1005***	−0.0637**	−0.0730**	−0.0920***	0.0243	−0.0416	−0.1374***
	[0.025]	[0.029]	[0.029]	[0.026]	[0.041]	[0.026]	[0.025]
Control*Reform	0.1144	0.1988	0.5020***	0.6142***	0.6341***	0.8821***	0.9465***
	[0.117]	[0.131]	[0.168]	[0.189]	[0.189]	[0.232]	[0.253]
Constant	0.3537***	0.3537***	0.3537***	0.3537***	0.3537***	0.3537***	0.3537***
	[0.006]	[0.006]	[0.006]	[0.006]	[0.006]	[0.006]	[0.006]
Observations	2682	2563	2520	2461	2433	2374	2335
R-squared	0.4285	0.4238	0.4177	0.4220	0.4073	0.4083	0.4079

Notes: This table presents the results of several difference-in-differences-in differences analyses obtained using the regressions described generally in eq. 2 and only using the subsample of firms operating in 9 states as the non-treated group. The dependent variable, Losses per Enrollee (LPE), is defined as the dollar amount of health insurance losses incurred by a given insurer, in a given state, during a given year, scaled by the number of plan enrollees for a given insurer, in a given state, during a given year. LPE is also scaled by 1000. In the table, “DDD estimator” is the difference-in-differences-in-differences estimator, “Treatment dummy” indicates firms operating in Texas, and “Reform Dummy” indicates years following the enactment of the Texas reform measures. Each column of output represents a separate analysis that compares LPEs in the year 2002 to a given, single year in the future. Clustered standard errors are presented in parentheses and ***indicates *p* < 0.01, **indicates *p* < 0.05, and *indicates p < 0

As a whole, the results of the DDD analysis provide additional support for the conclusion drawn in the DD analysis. There is very little evidence to suggest that the Texas tort liability reforms had a substantial prolonged spillover effect on health insurers operating in Texas in the years following the reforms. We do find some evidence that mean LPE in Texas increased at a greater rate than that of New Jersey, but this result does not hold for any of the four other subsamples of insurers used as controls. In addition, we find some evidence that LPE in Texas increased to a greater degree than non-Texas insurers in the year immediately following the reform but this effect does not persist to other future years. As a result, we are unable to definitively reject the null hypothesis, as there does not appear to be sufficient evidence in favor of the alternative.

## Conclusion

Using a difference-in-differences (DD) analysis, we find evidence that the Texas tort reform measures enacted in 2003 had little influence on the levels of health insurance losses per enrollee incurred by Texas health insurers. We utilize several non-treated groups and find that this result is not sensitive to the selection of the non-treated group. In an effort to control for state-specific insurance climates in general, we also consider automobile physical damage losses incurred by insurers in our sample and employ a difference-in-difference-in-differences (DDD) analysis, the results of which are largely consistent with the DD analysis. Our results provide support for our null hypothesis that reform measures in the medical malpractice market did not have a significant, persistent effect on health insurance losses.

Interestingly, our analysis does provide some evidence that the reforms had an immediate, but short-term spillover effect on health insurance markets. In particular, we find that the LPE for Texas insurers increased to a greater degree than non-Texas insurers in the first two years following the enactment of the reform. This evidence is consistent with the work of Paik et al. [22], who present evidence that Texas’ tort reform did not bend the healthcare cost curve downward as was suggested by many proponents of tort liability reform. However, since we find no other evidence that the reforms influenced the levels of health insurance losses incurred by Texas insures after 2005, our conclusion is that reforming the malpractice environment has largely insignificant economic implications for health insurance markets.

Our analysis provides novel and valuable insight into the consequences of tort reform. Proponents of tort liability reforms often suggest that reforms reduce defensive medicine practices, thereby reducing healthcare costs. Our analysis suggests that, if there are any persistent effects of tort reforms on provider-patient interactions, they do not spillover into health insurance markets. If anything, our analysis suggests these reforms may lead to initial, short-term increases in costs borne by health insurers. As such, our analysis should suggest to policy makers that, while there are potentially many economic benefits to tort liability reforms, reforms do not appear to be useful for influencing outcomes in health insurance markets.

An important consideration when interpreting our results is that our analysis provides evidence that the Texas reform had little influence on levels of health insurance losses, *in the aggregate*, across a variety of patient groups and provider specialties. That is, because health insurance companies reporting to the NAIC engage in a variety of health insurance lines, such as individual and group comprehensive healthcare, dental and vision, Medicaid, Medicare, and Federal Employee Health Benefits, our analysis captures the net result of changes in medical professionals’ behavior among a heterogeneous group of provider and patient types. This degree of heterogeneity is often not present in studies of malpractice liability’s influence on physician behavior and healthcare costs (for example [1, 2, 22]). As a result, if tort reform’s effects on provider behavior differ by the provider’s specialty type or the type of patient, then different analyses presented in the literature, utilizing different but relatively homogenous samples of provider or insured types, may yield conflicting results regarding the influence of malpractice exposure on healthcare cost and health insurance markets. Further research may consider how specific provider specialties and patient groups are influenced by changes in medical malpractice liability exposure.

## Footnotes

1 Reasons for the disagreement in the literature could arise from a number of sources including differences in sample characteristics, time periods, or econometric methods. As such, the additional evidence on the influence of malpractice exposure on the frequency/cost of services rendered by medical providers, presented in our analysis, is a valuable contribution to the ongoing debate in the literature.

2 For example, using data from the Physician Insurance Association of America (PIAA), Kessler and McClellan [24] find evidence that direct malpractice reforms reduce defensive medicine practices but do not influence health outcomes. Baicker, Fisher, and Chandra [25] find a positive relation between Medicare spending, especially on imaging services, and malpractice awards which provides support for the hypothesis that malpractice awards drive defensive medicine rates.

3 For example, Mello et al. [26] provide evidence that suggests physicians reduce or eliminated “high risk” aspects of their practice. Kessler, Sage, and Becker [27] provide evidence that tort reforms increase the supply of physicians.

4 This is not a universal sentiment in the literature. For example, Morrisey, Kligore, and Nelson [4] do not find any evidence that damage caps reduce the cost of employer sponsored health insurance. Given somewhat conflicting results in prior literature, our analysis is valuable in that it provides insight into the ongoing debate regarding how the medical professional liability climate ultimately influences the health insurance market.

5 In 2008 ten plaintiffs filed a federal lawsuit claiming the state’s non-economic cap violates the U.S. Constitution. The suit, similar to those filed in other states with such caps, argued that the cap has a direct impact on an injured patient’s potential jury award and, consequently, influences the value of filing the suit in the first place. A federal judge ruled that the cap was constitutional in 2012.

6 Interestingly, Hyman et al. [28] evaluate physician supply in Texas before and after the 2003 reforms. They find that physician supply in Texas was not deteriorating before 2003 and “did not measurably improve after the reform” (p.203).

7 Other Texas-specific studies include Friedson and Kniesner [29], who examine how the reform – specifically the non-economic damages cap – has affected pre-trial settlement speed and settlement amounts. They find that even though injured plaintiffs are compensated more quickly after reform, they receive a lower settlement. In addition, Paik, Black, Hyman, Sage, and Silver [20] evaluate the influence of the Texas reform on elderly patients. They find that tort reform strongly affected claim rates and payouts for all patients, but elderly claimants receive disproportionately lower payouts after reform. Further, a recent review of the Texas market indicates, among other things, that “the reform bill’s most significant achievements have been increased access to health care and an unanticipated positive economic impact on the Texas economy. By the end of 2013, 10 years and three months after the effective date of HB4, the number of licensed physicians in the state will almost have doubled” [19].

8 Paik et al. [22] posit a similar hypothesis regarding physicians’ perception of malpractice risk.

9 While much of our analysis relies on the NAIC’s health insurance database, we also utilize the property-casualty database for private passenger automobile loss data. Both databases also contain information relating to overall company financial information (for example, assets, liabilities, organizational form). However, the analysis presented here relies strictly on data from the state pages.

10 The dataset includes financial data filed with state insurance departments for all insurers classified as health insurers and much of the data, including premiums, claims, and enrollment, are reported separately for each state in which an insurer operates

11 Specifically, we restrict our sample to firms with at least $100,000 in direct premiums written, $100,000 in direct losses incurred, and 1000 enrollees. The inclusion of these filters does not substantially reduce our sample size and helps to reduce the effects of outliers in our analysis. These filters ensure that the insurers included in our analysis are non-trivial market participants in a given state that are likely to be influenced by the operational and regulatory climate of a given state.

12 This filter effectively ensures that we analyze firms that were operating before and after the Texas reform took effect.

13 Previous studies similar in nature to ours (for example, [22]) evaluate relative spending levels scaled by enrollees. In addition, because the size of health insurance markets, in terms of premium levels and insurers vary widely across states, it would be inappropriate to evaluate raw, unscaled loss levels across states. Further, a loss measure scaled by premiums is more indicative of financial/operational performance of the health insurer while losses scaled by plan participants (enrollees) better quantifies the amount of claims that insurance companies incur due to patients’ interactions with medical providers. We therefore believe that scaling by enrollees is the most appropriate method for our analysis.

14 The NAIC Health Insurance database, due to changes in reporting requirements, does not provide consistent and reliable data that can be used in our study before 2001. As such, we are forced to limit the start of our sample to 2001.

15 Such DD analyses are frequently used in the economics, finance, and insurance literature (for example, [22, 30, 31]). The method involves computing the difference between the pre- and post-, within-subjects differences of the treatment and control groups. As it applies to our analysis, the subjects are a sample of health insurance companies, the treatment group is a sub-sample of health insurers operating in Texas, and the non-treated group is a sub-sample of health insurers operating in other states.

16 Health insurers operating in Florida, Georgia, Illinois, Mississippi, Nevada, Ohio, Oklahoma, Oregon, and South Carolina are not included in this subsample.

17 These 18 states are Alabama, Arizona, Arkansas, Connecticut, Delaware, Iowa, Kentucky, Maine, Minnesota, New Hampshire, New York, North Carolina, Pennsylvania, Rhode Island, Tennessee, Vermont, Washington, and Wyoming. Paik et al. [22] also include the District of Columbia but, due to the unavailability of certain demographic data, we are forced to omit insurers operating there in our analysis.

18 These 9 states are Alabama, Arizona, Arkansas, Iowa, Kentucky, Minnesota, Tennessee, Washington, and Wyoming.

19 We recognize that some of the states in these subsamples enacted other reforms relating to medical malpractice during our sample period but, following Paik et al. [22], we believe these other reforms are not likely to cause major changes in losses per enrollee and are confident in the validity of these samples.

20 Studies such as Bertrand, Duflo, and Mallainthan [32] and Donald and Lang [33] suggest that, in certain cases, econometric issues lead researchers to incorrectly reject the null hypothesis of the statistical significance of a difference-in-differences coefficient. These studies provide evidence that clustering standard errors helps reduce the likelihood of a false rejection of the null. All results reported throughout the paper are from models with standard errors clustered at the firm level. In unreported results, we also implement another procedure found by Bertrand et al. [32] to reduce the likelihood of an inaccurate rejection of the null, the block bootstrap procedure. Our main results remain unchanged when the block bootstrap procedure is implemented. Further, our main results remain unchanged when we cluster standard errors by state and also when we include state and year effects (instead of the *Treat* and *Reform* dummies in the main specification). Therefore, we do not believe that the common econometric pitfalls of DD analysis described in the literature bias our results.

21 For example, changes in the number of people with health insurance.

22 We select private passenger automobile physical damage insurers because this line of business strictly relates to property losses. As such, factors associated directly with medical malpractice tort reform (for example, liability lawsuits, physician behavior, and the like) do not influence loss levels in this line of business. Similarly, factors associated directly with health insurance (doctors, healthcare costs, and so on) also do not influence loss levels in this line of business. Thus loss levels in private passenger automobile physical damage insurance are an appropriate control.

23 More specifically, we assign each insurer a number of automobiles based on proportion of premiums insurer *i* writes relative to all other insurers in a given state in a given year. For example, assume total premiums written in Texas for all insurers in 2010 was $1 billion and Insurer A wrote $100 million in premiums in 2010. If there are 1 million automobiles in the state of Texas in 2010, then Insurer A would be assigned (100 million/1 billion) X 1 million automobiles. Therefore, LPA for Insurer A would be incurred automobile physical damage losses divided by 100,000.

24 Note the “DD Estimator” coefficients and statistical significances reported are the regression coefficients of *Treat* ^∗^ *Reform* from equation 1. However, in this output, *Reform* takes the value of 0 if the year is 2002 and 1 if the year is either 2004, 2005, 2006, 2007, 2008, 2009, or 2010, depending on the specification.

25 These results are available from the authors upon request.

26 For the same reasons described in a previous footnote, all reported results are from models with standard errors clustered at the firm level. In addition, all reported results are robust to different clustering strategies described in a previous footnote as well as various combinations of state-level control variables.

27 These are the same control variables included in the DD analysis which are defined in a previous footnote. In addition, unreported analysis indicates that our main result is robust to the inclusion of additional/alternative demographic controls, such as uninsured rates and educational attainment.

28 Note the “DDD” estimates and statistical significances reported in the table are the regression coefficients of *Treat***Control*Reform* from equation 2. However, in this output, *Reform* takes the value of 0 if the year is 2002 and 1 if the year is either 2004, 2005, 2006, 2007, 2008, 2009, or 2010, depending on the specification.
